# Relatively Low Level of Antigen-specific Monocytes Detected in Blood from Untreated Tuberculosis Patients Using CD4^+^ T-cell Receptor Tetramers

**DOI:** 10.1371/journal.ppat.1003036

**Published:** 2012-11-29

**Authors:** Yuhong Huang, Yan Huang, Yimin Fang, Juan Wang, Yan Li, Nan Wang, Jianbo Zhang, Ming Gao, Lirong Huang, Fangfang Yang, Cong Wang, Shuxian Lin, Yanan Yao, Liangliang Ren, Yi Chen, Xuanjing Du, Dan Xie, Rongshun Wu, Kouxing Zhang, Lifang Jiang, Xinbing Yu, Xiaomin Lai

**Affiliations:** 1 Department of Microbiology, Zhongshan School of Medicine, Sun Yat-sen University, Guangzhou, China; 2 Key Laboratory of Tropical Diseases Control, Ministry of Education; Key Laboratory of Functional Molecules from Marine Microorganisms, Department of Education of Guangdong Province; Guangdong Provincial Research Center for Severe Infectious Disease Prevention and Control Technology, Zhongshan School of Medicine, Sun Yat-sen University, Guangzhou, China; 3 Department of Parasitology, Zhongshan School of Medicine, Sun Yat-sen University, Guangzhou, China; 4 Guangzhou Chest Hospital, Guangzhou, China; 5 The Third Affiliated Hospital of Sun Yat-sen University, Guangzhou, China; University of New Mexico, United States of America

## Abstract

The *in vivo* kinetics of antigen-presenting cells (APCs) in patients with advanced and convalescent tuberculosis (TB) is not well characterized. In order to target *Mycobacterium tuberculosis* (MTB) peptides- and HLA-DR-holding monocytes and macrophages, 2 MTB peptide-specific CD4^+^ T-cell receptor (TCR) tetramers eu and hu were successfully constructed. Peripheral blood (PBL) samples from inpatients with advanced pulmonary TB (PTB) were analyzed using flow cytometry, and the percentages of tetramer-bound CD14^+^ monocytes ranged from 0.26–1.44% and 0.21–0.95%, respectively; significantly higher than those measured in PBL samples obtained from non-TB patients, healthy donors, and umbilical cords. These tetramers were also able to specifically detect macrophages *in situ via* immunofluorescent staining. The results of the continuous time-point tracking of the tetramer-positive rates in PBL samples from active PTB outpatients undergoing treatment show that the median percentages were at first low before treatment, increased to their highest levels during the first month, and then began to decrease during the second month until finally reaching and maintaining a relatively low level after 3–6 months. These results suggest that there is a relatively low level of MTB-specific monocytes in advanced and untreated patients. Further experiments show that MTB induces apoptosis in CD14^+^ cells, and the percentage of apoptotic monocytes dramatically decreases after treatment. Therefore, the relatively low level of MTB-specific monocytes is probably related to the apoptosis or necrosis of APCs due to live bacteria and their growth. The bactericidal effects of anti-TB drugs, as well as other unknown factors, would induce a peak value during the first month of treatment, and a relatively low level would be subsequently reached and maintained until all of the involved factors reached equilibrium. These tetramers have diagnostic potential and can provide valuable insights into the mechanisms of antigen presentation and its relationship with TB infection and latent TB infection.

## Author Summary

*Mycobacterium tuberculosis* (MTB) is one of the most dangerous pathogens in the world. It is estimated that one-third of the world population contracts the bacteria during their lives. Approximately 5–10% of infected individuals will eventually develop an active form of the disease. Cellular immunity plays an important role in immunity against tuberculosis (TB); however, the host's defense mechanisms are not completely understood. Here, we developed a novel tool: MTB antigen-specific tetrameric CD4^+^ T-cell receptor (TCR) complexes that can detect MTB peptide-specific antigen presenting cells (APCs) in blood and local tissues. We found that a relatively low level of antigen-specific monocytes (i.e., APCs) was detected in peripheral blood (PBL) samples from untreated TB patients, and then increased to their peak levels during the first month after treatment, which probably had something to do with the decrease in APC apoptosis. Our research provides a new method for tracking dynamic changes in APCs that are associated with TB infection and latent TB infection, and an additional tool for the studies of TB immunity and its pathogenesis.

## Introduction

With approximately one-third of the world's population infected with *Mycobacterium tuberculosis* (MTB), tuberculosis (TB) continues to persist as a major infectious disease that significantly contributes to global morbidity and mortality [1]. However, 5–10% of infected individuals will eventually develop an active form of the disease. During TB infection, cellular immune responses are a critical part of the host's defense mechanisms [2]–[3]. Although the mechanisms of protection against TB are not completely understood, many studies have indicated the predominately protective role of CD4^+^ T cells [4]–[6]. MTB is endocytosed and survives in antigen-presenting cells (APCs), such as macrophages, monocytes, and dendritic cells. Some APCs present antigens in association with major histocompatibility complex (MHC) class II molecules that then stimulate CD4^+^ T cells. This process is essential to MTB infection [7], but the *in vivo* kinetics of APCs in patients with advanced and convalescent TB is not well characterized.

Many methods are available for studying the interactions between the T-cell receptors (TCR) on epitope-specific T cells and the epitopes and MHCs on APCs. Fluorescence-labeled, tetrameric MHC-peptide complexes have been widely used to detect and quantify antigen-specific T-cell populations *via* flow cytometry. Since Altman et al. first described the use of peptide/human leukocyte antigen (HLA) tetrameric complexes to directly visualize antigen-specific cytotoxic T lymphocytes (CTLs) using flow cytometry in 1996 [8], tetramerized MHC I and II complexes have been extensively used to quantify and characterize antigen-specific T cells [9]–[11] and probe TCR-MHC interactions. In 2004, Subbramanian et al. extended the tetrameric technique to TCR and successfully constructed high-affinity TCR tetramers [12]. In 2008, Wei H et al. developed γδ TCR tetramers in order to investigate the molecular mechanisms of the presentation of MTB-phospho-antigen to Vγ2Vδ2 T cells [13]–[14]. Soluble TCR tetramers have been utilized in a variety of functional assays, including the specific detection of target cells that have been pulsed with cognate peptide, discrimination between the quantitative changes that occur in antigen display at the cell surface, the identification of virus-infected cells, the inhibition of antigen-specific CTL activation, and the identification of cross-reactive peptides [13]–[19].

Until now, no MTB-specific CD4^+^ TCR tetramers have been reported. In the present study, we describe how we successfully constructed tetrameric CD4^+^ TCR complexes. Their binding specificities to monocytes obtained from peripheral blood (PBL) samples and macrophages in lung and lymph node sections from pulmonary TB (PTB) or lymph node TB patients and the inhibition of peptide-specific CD4^+^ T cells in PBL samples from patients with PTB were evaluated; In addition, any changes in tetramer-bound CD14^+^ monocytes from advanced and convalescent PTB outpatients were also tracked. MTB-specific TCR tetramers may provide a useful methods for detecting target cells and identifying specific, high-affinity interactions between HLA and peptides.

## Results

### Obtaining peptide-specific CD4^+^ T cells

In our previous studies, MTB peptides E6 and E7 from early secreted antigenic target-6 (ESAT-6) and C14 from culture filtrate protein-10 (CFP-10) were confirmed as HLA-DR-restricted and specific TCR ligands of CD4^+^ T cells, while C5 from CFP-10 was identified as a specific TCR ligand of both CD4^+^ T cells that is restricted by HLA-DR and CD8^+^ T cells by testing PBL and pleural fluid (PLF) samples from active TB patients using the IFN-γ-enzyme-linked immunospot (IFN-γ-ELISPOT) assay, lymphocyte-proliferation and -blocking tests, and intracellular cytokine staining (ICS). In this study, human MTB peptide-specific CD4^+^ T cells were obtained in order to access specific TCR tetramers. Mononuclear cells in PLF samples from patients with active tuberculous pleuritis were first analyzed using ELISPOT. Over 96% of the PLF samples reacted with the 4 peptides mentioned above, and 12–20% of enriched peptide-specific T cells were positively stained with the anti-CD4 monoclonal antibody (MAb). After separation of the CD4^+^ T cells using magnetic beads, the cells were stained with carboxyfluorescein succinimidyl ester (CFSE), and then allowed to proliferate *in vitro* by incubating them with the peptide for 9 days. After staining with anti-CD4-phycoerythrin (PE), >98% of the pure, expanded, peptide-responsive CD4^+^ T cells were obtained following cell sorting.

### Establishment of stable S2 cell lines that effectively express soluble CD4^+^ TCR and BirA

The CD4^+^ TCR α and β chain genes were successfully amplified from expanded peptide-responsive CD4^+^ T cells, each about 0.8 kb and 0.9 kb, respectively. Seventy-nine TCR α and β chain gene clones were isolated from 4 active TB patients. As shown in Table 1, 2 CD4^+^ TCR tetramers, eu and hu, were constructed using 2 different TCR α chains (e, accession number: HE862272 and h, accession number: HE862271; http://www.ebi.ac.uk/ena/) and the same TCR β chain (u, accession number: HE862270) that contained the high-frequency VDJ repertoire (AV12-3*01-J29*01/BV29-1*01-D2*01-J2-5*01 and AV1-2*01-J33*01/BV29-1*01-D2*01-J2-5*01) and complementarity-determining region 3 (CDR3) amino acid sequences (AMSARSGNTPLV/SLRDAKETQY and AVRDQNYQLI/SLRDAKETQY, respectively), which are the wild-type TCR α/β chains that were mainly cloned from subpopulations of C14- and E7-responsive CD4^+^ T cells that were obtained from an active TB patient (patient 11) with an HLA background of HLA-DRB1*1503/*1504 and HLA-DRB1*08032. Three other active TB patients shared the VDJ repertoire, the common CDR3 amino acid motifs of the α/β chains (AV12-3*01/AMSA of patient 10 with the TCR α chain of the eu-tetramer and AV12-3*01/AVRD of patients 5 and 10 with the TCR α chain of the hu-tetramer, respectively, as well as BV29-1*01/TQY of patient 9 and BV29-1*01/ETQY of patient 10 with the TCR β chains of the eu- and hu-tetramers, respectively), and HLA-DRB1 alleles (DRB1*150101 and DRB1*0818/*0806, DRB1*1503/*1504 and DRB1*03, and DRB1*1503/*1504 and DRB1*02023 in patients 5, 9 and 10, respectively).

**Table 1:** VDJ repertoire, CDR3 amino acid sequences of the α/β chains, and other related information regarding CD4^+^ TCR eu- and hu-tetramers.

Related information	V-D-J repertoire and CDR3 amino acid sequences of the α/β chains of CD4^+^ TCR tetramers			3 TB patients share the same VDJ repertoire and common CDR3 amino acid motifs of the α/β chains		
				Patient 5	Patient 9	Patient 10
CD4^+^ TCR tetramers	eu-tetramer	α chain	AV12-3*01-J29*01			AV12-3*01
			AMSARSGNTPLV			AMSA
		β chain	BV29-1*01-D2*01-J2-5*01		BV29-1*01	BV29-1*01
			SLRDAKETQY		…TQY	…ETQY
	hu-tetramer	α chain	AV1-2*01-J33*01	AV1-2*01		AV1-2*01
			AVRDQNYQLI	AVRD…		AVRD…
		β chain	BV29-1*01-D2*01-J2-5*01		BV29-1*01	BV29-1*01
			SLRDAKETQY		…TQY	…ETQY
TB patients' HLA-DRB1 alleles	DRB1*1503/*1504 and DRB1*08032 from patient 11			DRB1*150101 and DRB1*0818/*0806	DRB1*1503/*1504 and DRB1*03	DRB1*1503/*1504 and DRB1*02023
TB patients' CD4^+^ T cellular response to MTB peptides	The α chain of eu was cloned from peptide C14-responsive CD4^+^ T cells that shared peptides C5, E6, or E7-responsive CD4^+^ T cells with AV12-3*01/AMSA… or AV12-3*01/AM…; the α chain of hu was cloned from peptide E6-responsive CD4^+^ T cells that shared the peptide C14-responsive CD4^+^ T cells with AV1-2*01/…DSNYQSI; the β chain of eu and hu was cloned from peptide C14-responsive CD4^+^ T cells that shared peptides C5, E6, or E7-responsive CD4^+^ T cell with BV29-1*01/…TQY.			C14, C5, E6, and E7	C14, C5, E6, and E7	C14, C5, E6, and E7

The expression of monomeric TCR complexes in the culture supernatant of *Drosophila Schneider* 2 cells (S2 cells) was verified by detecting the corresponding tags. The target protein in the supernatant was purified by Ni-NTA agarose, and the purified sample was concentrated. Small aliquots of the purified samples were monitored using SDS-PAGE, dot-blot and Western blot assays. These assays confirmed that about 60 kDa of the soluble, biotinylated TCR α/β monomer was obtained, similar to our previous study [20].

### Efficiencies and specificities of TCR tetramers binding with the different MTB peptide/HLA-DR molecules that are expressed in the S2 cell lines

A panel of the MTB peptide/HLA-DR molecules that are displayed in the S2 cell lines (i.e., previously constructed, artificial APC lines) were used to determine the affinity of the constructed TCR tetramers for different MTB-peptide/HLA-DR molecules. After 48 hours of induction using CuSO_4_, the cells were incubated with PE-labeled TCR tetramer at 4 °C for 20 minutes and analyzed using flow cytometery. In order to determine the expression of HLA-DR in each cell lines, limited anti-HLA-DR antibody (L243-fluorescein isothiocyanate [FITC]; BD Pharmingen, San Jose, CA, USA) was co-incubated with the cells. Because the TCR-MHC-peptide interaction can be competitively blocked by L243, the percentage of tetramer positivity does not represent the tetramer-positive staining of all HLA-DR-peptide complexes, but instead reflects the affinity of TCR tetramers for different HLA-DR-peptides.

**Figure 1:**
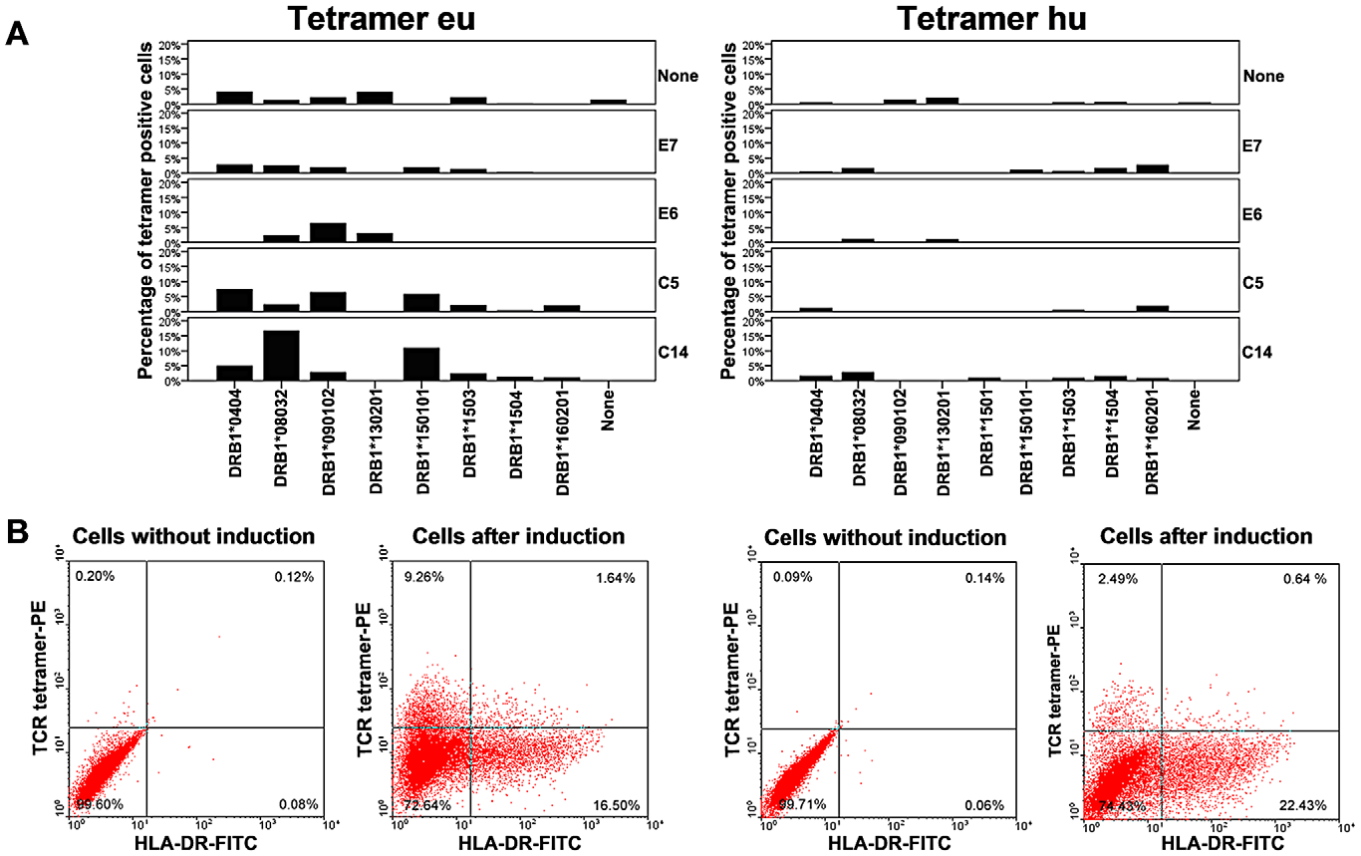
Flow cytometric analysis of the binding of TCR tetramers to artificial APCs. (A) The X-axis indicates APCs with different HLA-DR alleles and Y-axis indicates the percentages of tetramer-positive APCs induced by different MTB-specific peptides (E7, E6, C5, and C14). On the X-axis, “none” indicates non-induced cells or cells that only expressed HLA-DR without the peptide listed on the Y-axis. (B) Examples of dot plots showing the binding of TCR tetramers with artificial APCs. Only the background stainings of the non-induced cells were shown (0.32% for eu and 0.23% for hu). Positive stainings by either the tetramers in cells expressing C14/HLA-DR following induction (10.90% in C14/HLA-DRB1*150101 stained with eu and 3.13% in C14/HLA-DRB1*08032 stained with hu), were observed. The expression of HLA-DR was confirmed by staining with the anti-HLA-DR antibody (L243-FITC). Because the binding of the TCR tetramers to peptide/HLA-DR complexes could be competitively blocked by L243, small cells appeared in the upper right quadrant of the dot plots.

TCR tetramers were able to bind to MTB peptide C14/HLA-DRB1*08032 displayed in S2 cells, while only background staining was accomplished in cells without induction (Figure 1B). The positive-detection rates (Figure 1A) of eu-tetramer staining in the cell lines that expressed peptides C14/HLA-DRB1*08032, C14/HLA-DRB1*150101, C5/HLA-DRB1*0404, E6/HLA-DRB1*090102, C5/HLA-DRB1*090102, and C5/HLA-DRB1*150101 on the cell membrane were 18.65%, 10.90%, 7.30%, 6.40%, 6.32%, and 5.71% after induction, respectively. As for the hu-tetramer, the rates were <2.7%, except in C14/HLA-DRB1*08032 (3.13%) and E7/HLA-DRB1*160201 (3.12%). The 2 tetramers did not react with non-induced cells or cell lines that only expressed the HLA-DR molecules (Figure S1).

### TCR tetramers could specifically inhibit the proliferation of MTB peptide-induced CD4^+^ T cells

CD4^+^ T cells can be activated when TCRs on the cells were occupied by immunogenic peptide bound to an HLA II molecule, together with a co-stimulatory signal from the APC. Activation leads to cell proliferation which can be identified using CFSE T cell proliferation assay. Along with the cells that are labeled with CFSE on day 0, upon cell division each CFSE-high cell will lose half of its CFSE labeling, so that the populations of CFSE-low daughter cells can be visualized using flow cytometry. On the other hand, when co-incubated with peptide-specific TCR tetramer, the tetramer competitively inhibits the binding of peptide-HLA to the TCR on CD4^+^ T cell. As a result, the proliferation of CD4^+^ T cells is suppressed.

**Figure 2:**
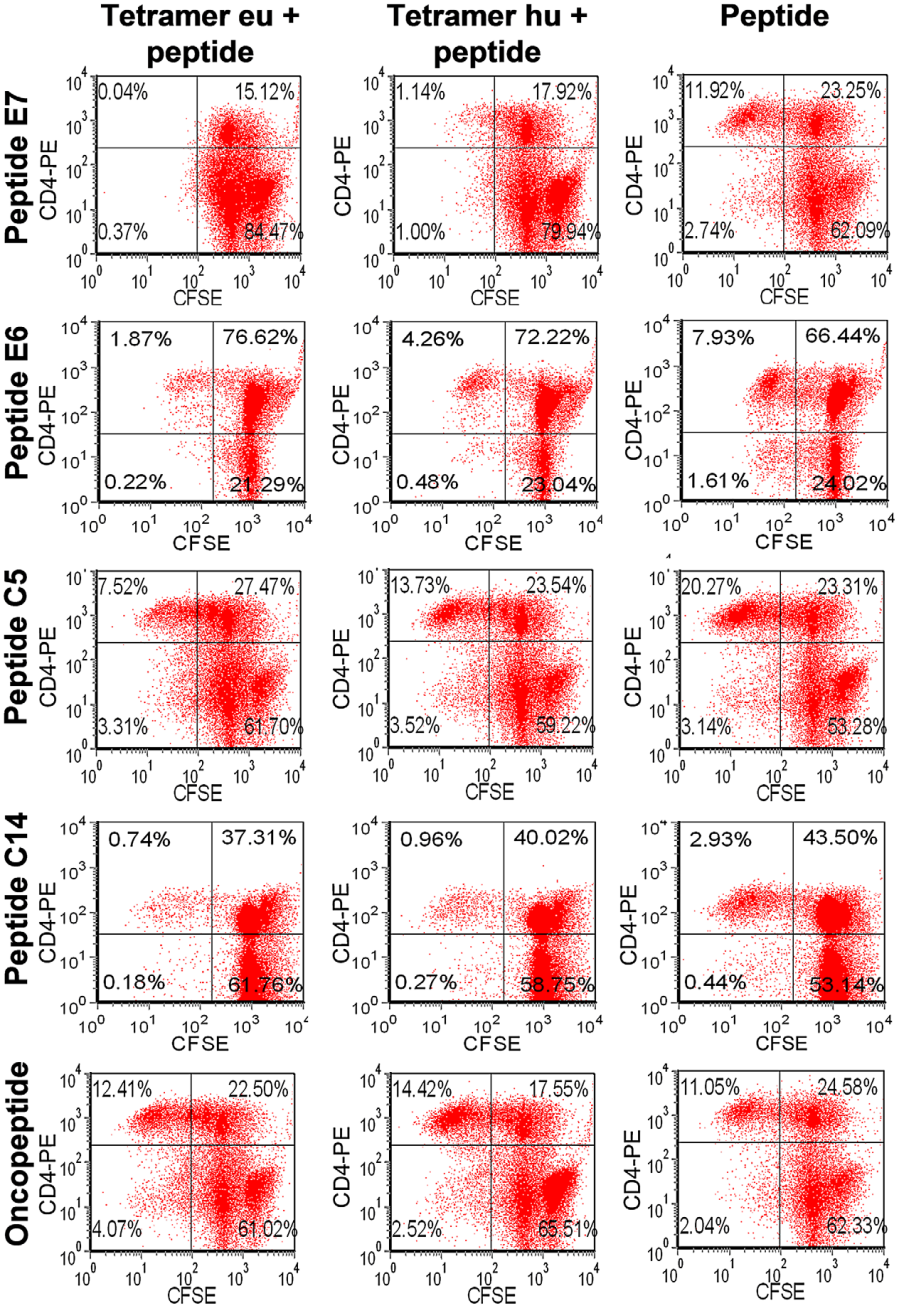
Proliferation of TCR tetramer-treated and -untreated PBMCs. After 9 days of cultivation, CFSE-labeled PBMCs were stained with PE-labeled CD4 MAbs and analyzed by flow cytometry in order to determine their proliferation activities. Determination of the divided cells was based on the low-CFSE fraction. The results show that peptides E7, E6, C5, and C14 and oncopeptide promoted the proliferation of CD4^+^ T cells (the third line). When loaded with the eu- or hu-tetramer, the percentage of low-CFSE CD4^+^ T cells treated with peptides E7, E6, C5 or C14 decreased (the first and the second row) but not cells treated with oncopeptide.

A single dose of TCR tetramer was added to the peripheral blood mononuclear cells (PBMCs) that were co-cultured with peptide on day 0. After 10 days of culturing, the divided (i.e., low CFSE fluorescent) CD4^+^ T cells were quantified. As shown in Figure 2, the percentage of low-CFSE CD4^+^ cells was significantly lower in cells cultured with TCR tetramer and peptide than cells cultured with only peptides E7, C5, E6, and C14, respectively. However, there were no significantly differences between the cells incubated with or without TCR tetramer when the cells were stimulated with oncopeptide. This indicates that the eu- and hu-tetramers inhibit, to various degrees, the proliferation of CD4^+^ T cells that is induced by peptides E7, C5, E6, and C14, respectively, but do not inhibit oncopeptide-induced CD4^+^ T cells proliferation.

Above all, the results show that the 2 pure, MTB-specific CD4^+^ TCR tetramers could be used as staining reagents to analyze tetramer-bound (CD14^+^) APCs in clinical samples.

### Tracking tetramer-bound CD14^+^ monocytes in advanced PTB inpatients and convalescent PTB outpatients

**Figure 3:**
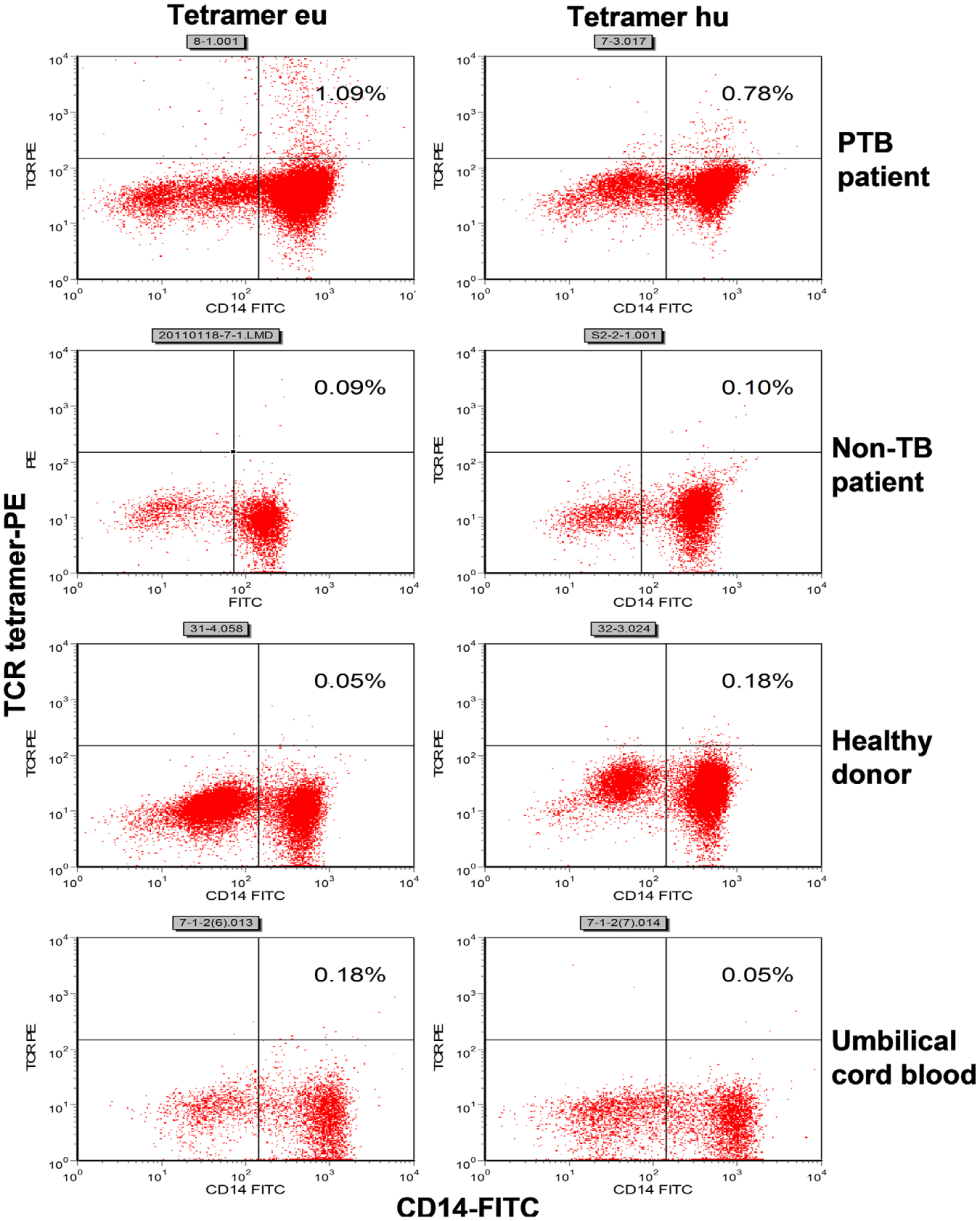
Flow cytometric analyses of PMBCs stained with the 2 TCR tetramers. PMBCs in each group were isolated from the same donor and monitored by staining with the eu- or hu-tetramers. The percentages of tetramer-bound CD14^+^ cells in the PMBCs that were isolated from PBL sample of an active PTB inpatient by eu- or hu-tetramer staining were 0.78% or 1.09%, respectively. The percentages of tetramer-bound CD14^+^ cells in the PMBCs isolated from umbilical cord blood, PBL samples collected from non-TB patient and healthy donor were all <0.18%.

**Figure 4:**
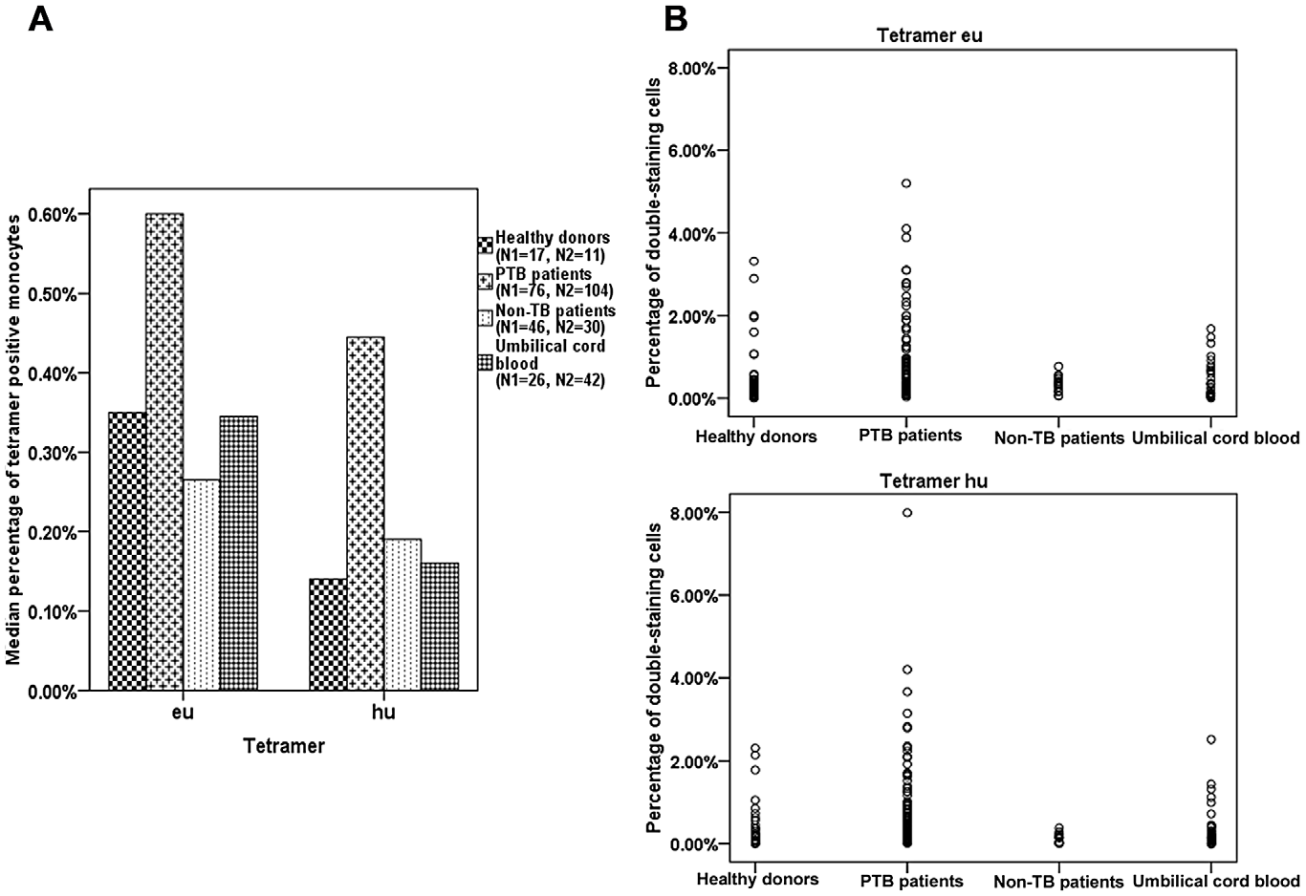
Statistical analyses of the tetramer-bound CD14^+^ monocytes detected in PBL samples from active PTB inpatients. The percentage of tetramer-bound CD14^+^ monocytes was detected by flow cytometry. (A) The clustered bar graph shows that the median percentages of tetramer-bound CD14^+^ monocytes in the PBL samples from PTB inpatients (second bar; 0.6% and 0.45%) were significantly higher than those of the control groups (Mann-Whitney U test, *p*<0.01). N1 and N2 indicate the number of samples stained with the eu- and hu-tetramer in each group, respectively. (B) The scatter graphs show that a high percentage of tetramer-bound CD14^+^ monocytes was only observed in PBL samples from PTB inpatients following staining with both tetramers.

In 76 active PTB inpatients, a median of 0.60% (range: 0.26–1.44%) of the CD14^+^ monocytes was positively stained with the eu-tetramer, while a median of 0.45% (range: 0.21–0.95%) was positively stained with the hu-tetramer in 104 active PTB inpatients. Some positively stained CD14^+^ monocytes were detected in a few samples from the healthy donor and umbilical cord blood groups, though there were definite and significant differences in the median percentages of tetramer-bound CD14^+^ monocytes between the PTB patient group and each of the control groups (*p*<0.01), as determined using the Mann-Whitney U test (Table 2; Figures 3 and 4A [clustered bar graph]). The actual distribution of the percentage of tetramer-bound CD14^+^ monocytes in each sample is shown in Figure 4B (scatter graphs). A few positive stained samples were found in the healthy donor group, which may have been related to latent TB infection. Nonetheless, high tetramer-positive samples were only apparent in the PTB patients.

**Table 2:** Percentage comparisons of tetramer-bound CD14^+^ monocytes in blood samples from the PTB inpatient group and 3 control donors groups by eu- and hu-tetramer staining and flow cytometric analysis.

Groups	eu-tetramer				hu-tetramer			
	N	P25	P50	P75	N	P25	P50	P75
PTB inpatients	76	0.26	0.60	1.44	104	0.21	0.45	0.95
Non-TB inpatients	46	0.12	0.27	0.42	30	0.06	0.19	0.58
Healthy donors	17	0.18	0.35	0.48	11	0.02	0.14	0.24
Umbilical cord blood	26	0.12	0.35	0.76	42	0.08	0.16	0.40

N, sample size; P25, P50, and P75 indicate the lower, median, and upper quartiles of the percentage of tetramer-bound CD14^+^ monocytes, respectively. The Mann-Whitney U test shows that there were significant differences in the median percentages of tetramer-bound CD14^+^ monocytes, as determined using either of the tetramer tests, between the PTB patient group and each of the control groups, respectively (*p*<0.01).

The PBL samples, which either demonstrated a high affinity for TCR tetramers or negative double-labeling staining to both tetramers, were selected for HLA-DR typing. The results show that the eu-tetramer has a high affinity for HLA-DRB1*13, *16, *11, *07, *14, and *15, while the hu-tetramer has a high affinity for HLA-DRB1*16, *14, *09, *15, and *04 (Table 3). These results indicate that the 2 TCR tetramers interact with multiple HLA-DR molecules. This is because these 2 tetramers consisted of TCR β chains with the same amino acid sequence and obtained from the same patient with an HLA-DRB1 background who shared some similar VDJ repertoires, common CDR3 amino acid motifs, and overlapping HLA-DRB1 backgrounds with other patients, as well as 2 similar α chains that consisted of the 2 TCR tetramers.

**Table 3:** HLA-DRB1 alleles of active PTB patients and the percentages of TCR tetramer-bound CD14^+^ monocytes in PBL samples.

HLA-DRB1 alleles	eu		hu	
	Cases	Positivity rate^a^	Cases	Positivity rate
DRB1*13	4	2.06	4	0.30
DRB1*16	3	1.85	3	0.95
DRB1*11	1	1.84	0	
DRB1*07	6	1.34	5	0.17
DRB1*14	4	1.04	7	0.68
DRB1*15	10	0.98	9	0.56
DRB1*12	6	0.70	8	0.32
DRB1*03	3	0.60	2	0.32
DRB1*08	3	0.57	4	0.19
DRB1*04	5	0.52	6	0.42
DRB1*09	9	0.44	10	0.58

a The positivity rate indicates the median percentage of double-stained-positive monocytes in PBL samples from active PTB patients.

**Figure 5:**
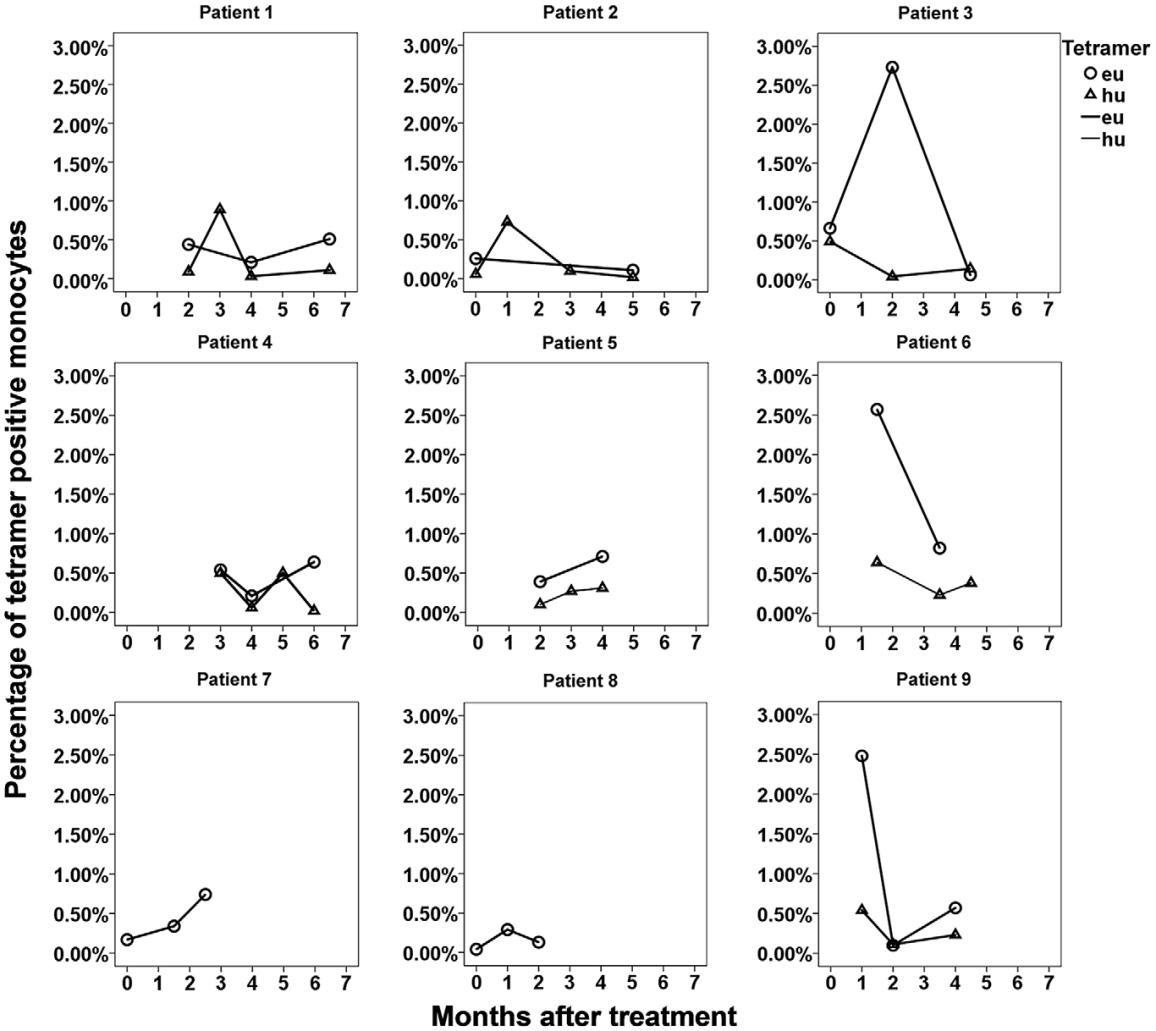
Dynamic changes in tetramer-bound CD14^+^ monocytes in PBL samples from 9 PTB outpatients during treatment. Continuous time point-tracking of the PBL samples obtained from 9 active PTB outpatients during 6 months of regular anti-TB treatment was performed in order to assess the percentage changes in TCR tetramer-bound CD14^+^ monocytes. Each point represents the percentage of eu- or hu-tetramer-stained positive monocytes determined by flow cytometry at the indicated month after treatment.

**Figure 6:**
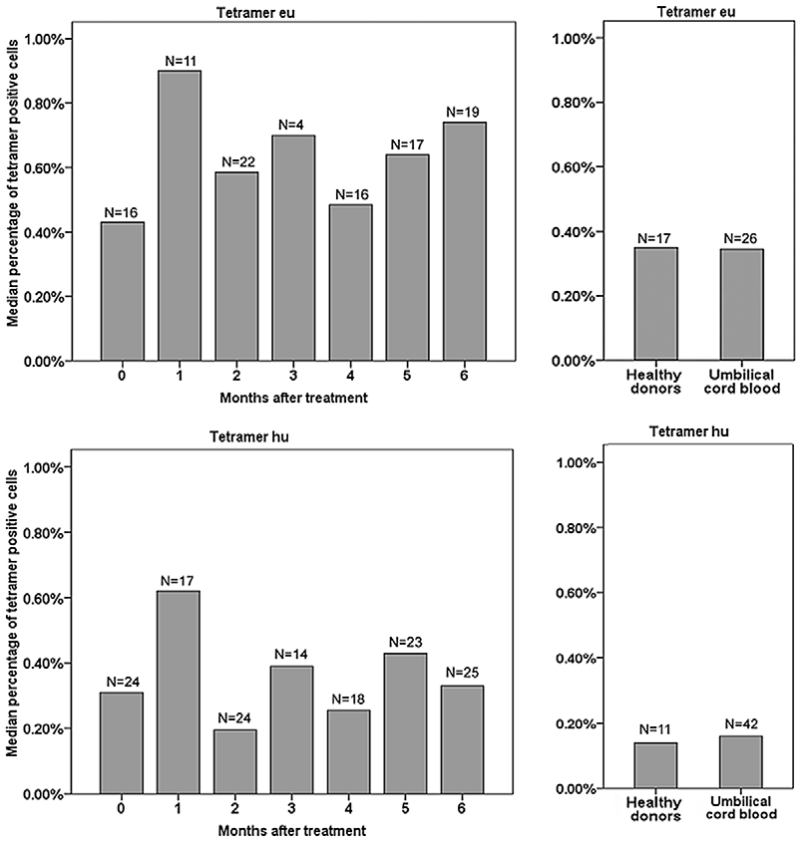
Percentage changes in tetramer-bound CD14^+^ monocytes in PTB outpatients in the continuous treatment group. PBL samples from 7 continuous time point-racked PTB outpatients, including patients who had not received treatment and those who received 6 months of regular anti-TB treatment, were stained with the eu- or hu-tetramer and analyzed using flow cytometry. N indicates the sample size of each group. After a month of treatment, there were statistical differences in terms of the percentages of tetramer-positive cells between PTB patients and both the healthy donors and umbilical cord blood groups (according to the results of the Mann-Whitney U test: 0.86%, 0.35% and 0.14%, *p*<0.001 and *p*<0.009 for eu-tetramer staining, respectively; and 0.62%, 0.14% and 0.16%, *p*<0.000 and *p*<0.000 for hu-tetramer staining, respectively). There was no significant difference between PTB patients at the indicated month after treatment in terms of the results of either tetramer staining according to the result of the Kruskal-Wallis H test (*p* = 0.585 and *p* = 0.141, respectively).

In a follow-up study, continuous time point-tracked PBL samples from 9 active PTB outpatients (Table 4 and Figure 5) were drawn every month during regular, 6-month-long, anti-TB treatments in order to assess the changes in the percentage of TCR tetramer-bound CD14^+^ monocytes using eu- and hu-tetramer staining and flow cytometric analysis. Figure 5 shows that the percentage changes in all patients followed roughly the same trends, except patient 7. In patients 2, 3, 6, and 9, along with the amendment of TB symptoms (although there were some small undulations), the median percentages were at first low before treatment, increased to their highest levels during the first month, and then began to decrease during the second month until finally reaching and maintaining a relatively low level after 3–6 months of treatment. These relatively low and small percentage changes were observed in patients 1, 4, 5, 7, and 8 and might be related to the different HLA backgrounds or immunity and disease statuses of the individual patients. Furthermore, group PBL samples from 7 continuous time point-tracked PTB outpatients groups, which included time points recorded before treatment and monthly samples obtained during regular 6-month-long anti-TB treatment periods, were detected using flow cytometry and eu- and hu-tetramer staining. Figure 6 shows that the median percentages of tetramer-bound CD14^+^ monocytes were 0.43%, 0.90%, 0.59%, 0.70%, 0.49%, 0.64%, and 0.74% at months 0, 1, 2, 3, 4, 5, and 6, respectively, according to eu-tetramer staining, and were 0.31%, 0.62%, 0.20%, 0.39%, 0.26%, 0.43%, and 0.33% according to hu-tetramer staining, respectively. Similarly, as shown in Figure 5 which depicts the continuous time point-tracking results of 9 active PTB outpatients, the median percentages were at first low before treatment, increased to their highest levels during the first month, and then began to decrease during the second month until finally reaching and maintaining a relatively low level after 3–6 months of treatment (although there was some undulation); however, all patients demonstrated relatively higher levels than the healthy donor and umbilical cord blood groups. As shown in Table 2, 0.35% and 0.35% of samples demonstrated positive eu-tetramer staining and 0.14% and 0.16% of samples demonstrated positive hu-tetramer staining, respectively. However, the Mann-Whitney U test determined that the statistical differences were mainly found between the treatment groups during the first month of treatment and both the healthy donors and umbilical cord blood groups (0.86%, 0.35% and 0.14%; *p*<0.001 and *p*<0.009 for eu-tetramer staining, respectively; and 0.62%, 0.14% and 0.16%; *p*<0.000 and *p*<0.000 for hu-tetramer staining, respectively); no significant differences were observed between any PTB groups in terms of the results of the either of the tetramer tests, as determined by the Kruskal-Wallis H test (*p* = 0.585 and *p* = 0.141, respectively). Because TCR tetramers are HLA II-dependent, only MTB-specific APCs with matching HLA background can be detected by the 2 TCR tetramers. Therefore, the differences between the positive rates of the samples from different treatment periods would be disguised.

**Table 4:** HLA-DRB1 alleles of 9 active PTB outpatients.

Patients' number	HLA-DRB1 alleles
1	HLA-DRB1*0301/DRB1*1302
2	HLA-DRB1*0401/DRB1*1303
3	HLA-DRB1*0701/DRB1*1405
4	HLA-DRB1*0406/DRB1*1502
5	HLA-DRB1*0403/DRB1*0403
6	HLA-DRB1*1501/DRB1*1602
7	HLA-DRB1*0301/DRB1*1502
8	HLA-DRB1*0803/DRB1*1405
9	HLA-DRB1*0701/DRB1*1101

### The apoptosis of MTB-infected monocytes is probably related to live bacteria and their growth, and decreases following drug treatment

**Figure 7:**
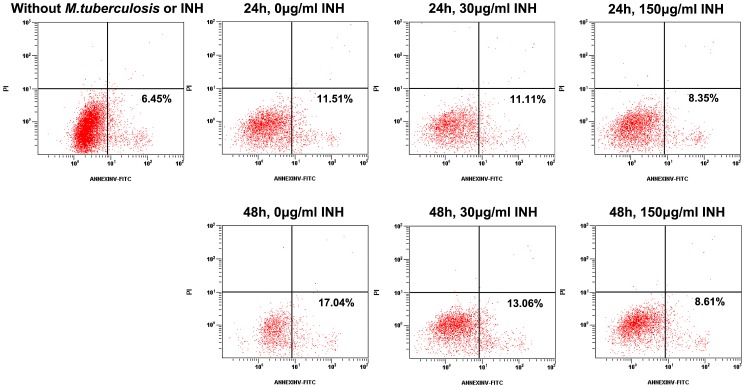
Apoptosis of MTB-induced THP-1 cells analyzed using flow cytometry with annexin-V-FITC/PI staining. THP-1 cells were co-cultured with 3×10^5^ MTB H37Ra. A single dose of INH was added. Cells incubated without MTB H37Ra or INH were served as the negative controls, while cells incubated with only MTB H37Ra were used as the positive control. The results show that INH could reduce or prevent MTB-induced apoptosis in THP-1 cells.

**Figure 8:**
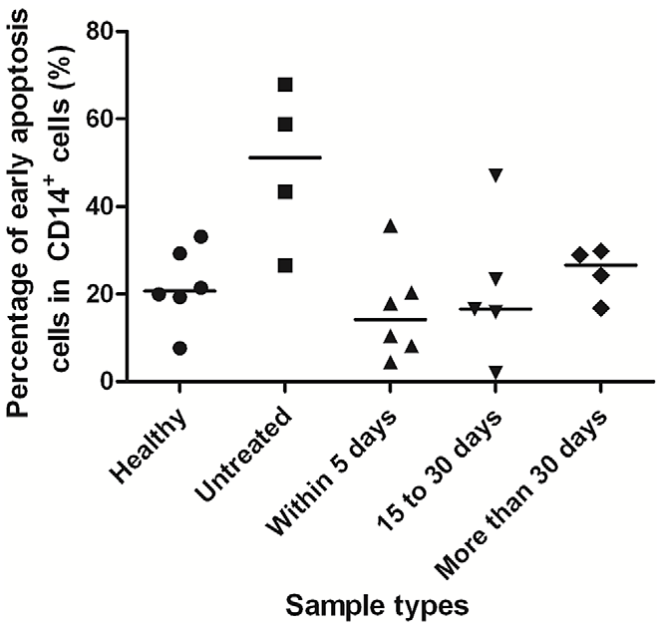
Percentage of early-apoptosis CD14^+^ cells from healthy donors or the continuously treated PTB patients. Apoptotic CD14^+^ cells were analyzed by flow cytometry using Annexin V plus PI staining *in vitro*. Annexin V^+^PI^−^ cells represent the early apoptotic populations. Healthy, healthy donors; untreated, untreated PTB patients; within 5 days, PTB patients who received regular anti-TB treatment within 5 days; 15 to 30 days, PTB patients received regular treatment for 15–30 days; More than 30 days: PTB patients received regular treatment for >30 days. The presented data indicate that the percentage of apoptotic cells dramatically decreased after treatment.

Earlier researches have reported that APC apoptosis is an important process in TB [21], [22]. We speculate that the relatively low level of tetramer-positive monocytes in blood from untreated TB patients is probably related to the apoptosis of APCs due to live bacteria and their growth; on the other hand, the increase in the first month after treatment may be related to the decrease in APC apoptosis. In order to verify these, MTB H37Ra-induced apoptosis of human acute monocytic leukemia cells (THP-1 cells) was examined by flow cytometry using Annexin V plus PI staining *in vitro*. The results show that the apoptosis of the THP-1 cells decreased following treatment with isoniazid (INH) (Figure 7). Analysis of CD14^+^ cells obtained from healthy donors, untreated or continuously treated PTB patients also showed that the percentages of apoptosis dramatically decreased following treatment (Figure 8).

### Detection of tetramer-bound and antigen-specific CD14^+^ macrophages in local TB lesions by *in situ* eu- and hu-tetramer staining and confocal laser-scanning microscopy

**Figure 9:**
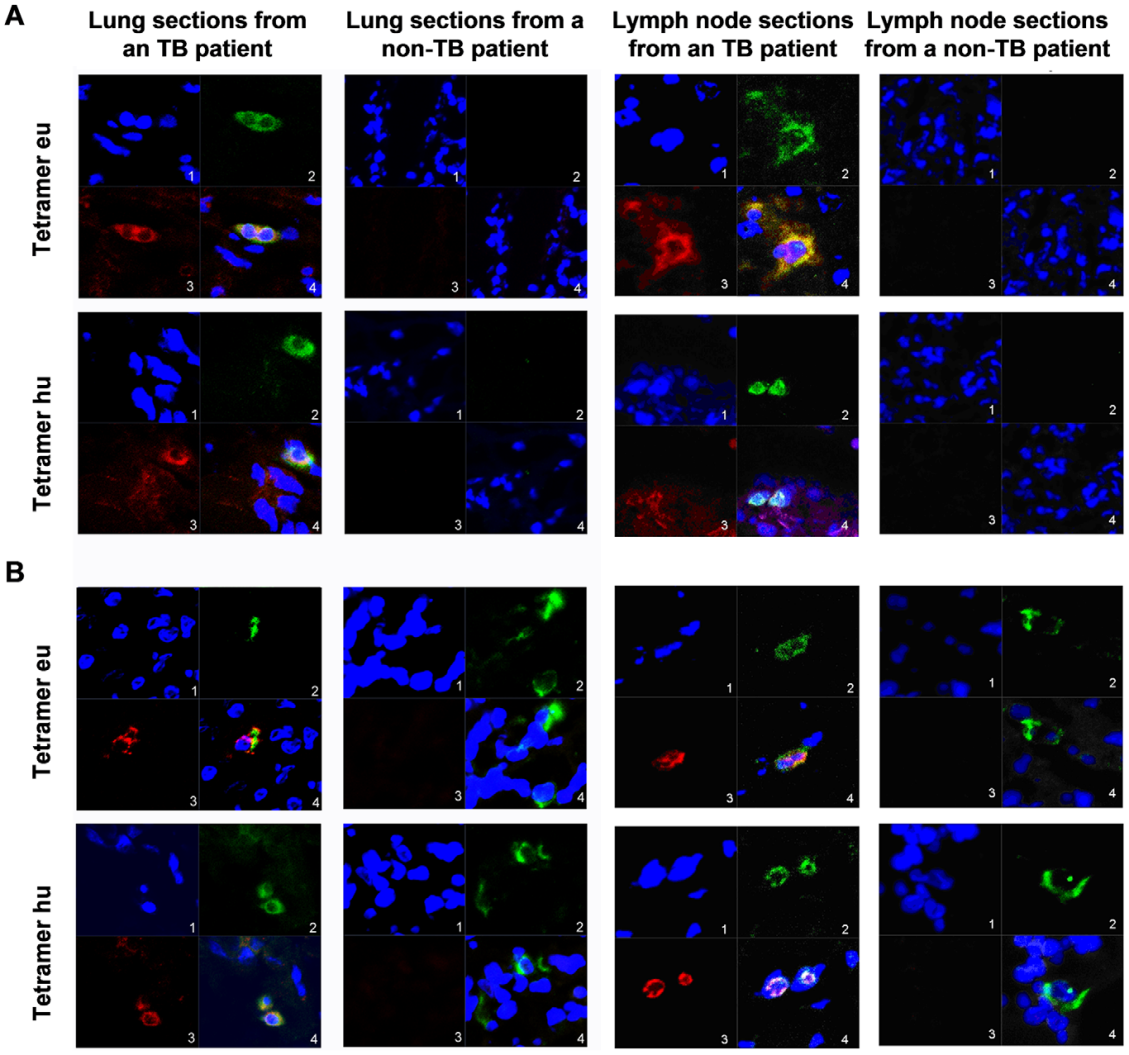
*In situ* detection of tetramer-bound MTB APCs using confocal laser-scanning microscopy. (A) The MTB antigen and peptide/HLA-DR were detected using *in situ* immunofluorescent staining with anti-MTB antibody and TCR tetramer, respectively. The nucleus is stained blue (panel 1), TCR tetramer is stained green (panel 2), and MTB antigen is stained red (panel 3). Panel 4 shows a merged image. (B) CD14 and peptide/HLA-DR were detected using i*n situ* immunofluorescent staining with anti-CD14 antibody and TCR tetramer, respectively. The nucleus is labeled in blue (panel 1), CD14 is labeled in green (panel 2), and the TCR tetramer is labeled in red (panel 3). Panel 4 shows a merged image. Lymph node and lung sections of active TB patients show co-staining in both (A) and (B), but no staining or only anti-CD14 antibody staining were observed in the lung and lymph node sections from non-PTB patients.

To determine the distribution and number of tetramer-bound and antigen-specific CD14^+^ macrophages in local TB lesions, fresh-frozen, 8-µm-thick sections of lung and lymph node from active TB patients were probed using the anti-MTB antibody and TCR tetramer or the anti-CD14 antibody and TCR tetramer, respectively, followed by nuclear staining with 4′, 6-diamidino-2-phenylindole (DAPI) and observation with confocal laser-scanning microscopy. The results of both staining strategies demonstrated double-positive stainings in the lung and lymph node sections from 2 active TB inpatients with HLA-DRB1*040601/DRB1*110103 and HLA-DRB1*1202/DRB1*1202 backgrounds, respectively, and negative responses to the TCR tetramers and anti-TB antibodies in the lung and lymph node sections of 2 non-TB patients with HLA-DRB1*1504/DRB1*1202 and HLA-DRB1*1202/DRB1*0406 backgrounds, respectively (Figure 9). These results suggest that there are TCR tetramer-bound and MTB antigen-positive CD14^+^ macrophages (i.e., APCs) in the local TB tissues.

## Discussion

In this study, we amplified TCR α and β chains from expanded peptide-responsive CD4^+^ T cells that were separated from ELISPOT-positive PLF mononuclear cells that were obtained from patients with active tuberculous pleuritis. High-frequency α and β chain families were analyzed and selected. The full-length TCRs were expressed and biotinylated using insect cells. The TCR heterodimers were purified and tetramerized. The affinities and specificities of the 2 selected TCR tetramers (eu and hu) for binding to MTB peptide/HLA-DR molecules were confirmed using a series of artificial APCs that expressed different MTB peptide/HLA-DR molecules in S2 cells.

Although CD4^+^ TCR chain sequences are highly diverse, we found that MTB peptide-responsive CD4^+^ T cells derived from different individuals shared the exact same TCR α and β chain sequences, CDR3 sequences, genetic families, or common CDR3 amino acid motifs. The TCR α/β chain families that were present at a high frequency in the PLF mononuclear cells that responded to the same or different MTB peptides were selected for the construction of TCR tetramers. As shown in Table 1, 2 peptide C14-responsive α chains from different CD4^+^ T-cells clones and 1 C14-responsive β chain from the same CD4^+^ T-cell clone (these chains share the same or similar VDJ repertoire and CDR3 amino acid motifs) were selected for the preparation of the eu- and hu-tetramers. Our data clearly demonstrate that these 2 tetramers are capable of recognizing MTB peptides in the context of multiple HLA-DR molecules, which is consistent with the results of earlier studies [23]–[25]. Meanwhile, both eu- and hu-tetramers could inhibit CD4^+^ T-cell proliferation at different levels, which is induced by a variety of peptides (E7, C5, E6, and C14). These results indicate the different detection efficiencies and specificities of these 2 tetramers. On the other hand, because of the limited numbers of cases enrolled in this study, we were unable to determine all of the HLA-DR alleles that interacted with the tetramers.

TB is characterized by the formation of local granulomas, caseous necrosis, and cavities where macrophages, their derived cells (e.g., Langhans-type multinucleated giant cells), and a variety of lymphocytes are recruited. Macrophages are believed to differentiate from the recruited monocytes in circulation. Both macrophages and monocytes are crucial cells involved in immune defense, in which bacteria grow and survive [26], [27]. To investigate MTB-specific APCs *in vivo* in patients with advanced and convalescent TB, we evaluated the tetramer-positive monocytes in a series of clinical samples using flow cytometry. Inpatients with advanced PTB, both those who were untreated and those who had just begun treatment, were recruited to participate in the present study. In their PBL samples, the percentages of tetramer-bound CD14^+^ monocytes ranged between 0.26–1.44% and 0.21–0.95% by according to the results of eu- and hu-staining respectively. The percentage of tetramer-positive cells was significantly higher than those measured in samples obtained from non-TB patients, healthy donors, and the umbilical cord groups. The 2 tetramers could also specifically detect macrophages *in situ* in lungs and lymph nodes sections from untreated and advanced TB patients using immunofluorescentce staining.

Surprisingly, continuous time-point tracking of the 2 tetramers in the PBL samples obtained from active PTB outpatients undergoing treatment demonstrated that the median percentages were at first low before treatment, increased to their highest levels during the first month, and then began to decrease during the second month until finally reaching and maintaining a relatively low level after 3–6 months of treatment; however, all demonstrated relatively higher levels than the healthy donor or umbilical cord groups. Higher detection rates were measured in the PBL samples obtained from the PTB inpatients group than samples obtained from the PTB outpatients group before anti-TB treatment. This may have been due to the fact that the former group contained some patients in the initial treatment stage (usually within the first 20 days of treatment). Also, the inpatients were sicker than the outpatients and carried more MTB. These results suggest that there is a relatively low level of MTB-specific monocytes in the circulation of advanced and untreated PTB patients. The quantity of MTB-specific monocytes is probably related to the local recruitment of APCs, focus formation, and APC apoptosis or necrosis due to live bacteria and their growth. Earlier studies have reported that APC apoptosis is an important process and major event that is necessary to produce caseous necrosis in granulomas and other lesions during mycobacterial infection [21], [22] and is associated with live mycobacteria and mycobacterial molecules [28]–[39]. On the other hand, MTB can also induce macrophage necrosis by inhibiting the repair of plasma membranes [40]. Placido et al. found that by using a virulent strain MTB H37Rv, apoptosis was induced in a dose-dependent fashion in macrophages that were obtained by broncho alveolar lavage from patients with TB [41]. In our research, when THP-1 cells were co-cultured with MTB H37Ra *in vitro*, apoptosis decreased when INH was added. In addition, we found that early apoptosis of CD14^+^ cells dramatically decreased after treatment. Because APC apoptosis decreased, the quantity of APC increased. Also, during the early stages of treatment, bacteria are killed or a large amount of MTB peptides are picked up and processed by APCs, which expands the T-cell population [42]. That, in turn releases IFN-γ, other cytokines and lymphokines then activate macrophages. Pedroza-Gonzalez et al. found that CD14^+^ cells were recruited into lungs by day 14 after MTB infection, significantly increased by day 21 (approximately 16-fold over the control group), and elevated during the chronic phase of infection [43]. So, the decrease in the local recruitment and consumption of APCs and the bactericidal effects of anti-TB drugs would induce a peak in the quantity of monocytes for a short period of time during the first month and, subsequently, a relatively lower level would be reached and maintained due to equilibrium between the various factors involved. Perhaps in the later periods of treatment the peptide levels would decrease to the background level along with the clearance of bacteria.

In our data, although the frequency of TCR tetramer-positive cells in PBL samples was low, the frequency of positive cells in the artificial APCs was high (up to 18.65%). This implies a high affinity for the tetramers. Although there are no data on the frequency of TCR tetramer-positive cells in TB patients, low levels of peptide/MHC tetramer-positive cells in PBL samples obtained from TB patients have been reported in many studies [44]–[46]. In addition, low numbers of peptide/MHC II tetramer-positive cells have been reported in recent studies on the detection of CD4^+^ T cells in response to infectious agents, autoantigens, allergens and tumour antigens, with frequencies generally ranging from 0.02 to 0.6% of the total number of CD4^+^ T cells [47]–[54].

The low frequency of tetramer-positive cells may be due to the fact that most tetramer-staining studies on humans have relied on the enumeration of the T-cell populations that are present in circulating PBL not at the primary site of inflammation. Higher frequencies probably exist in the compartments that are more directly affected by the immune response of interest. Meyer et al. found a nearly 33-fold increase in the abundance of outer-surface protein A-specific CD4^+^ T cells at the primary site of inflammation [55]. Mice infected with the sendai virus demonstrated a remarkably high frequency (13%) of activated CD4^+^ T cells in the lung sections using specific MHC-immunoglobulin multimers [56]. Moreover, in PBMCs obtained from TB patients or MTB-infected animals, the 10-fold expansions of peptide/HLA-DR tetramer-bound epitope-specific CD4^+^ T cells were seen after specific peptide stimulations *in vitro* [20], [44].

Because the affinities of the CD4^+^ TCR tetramers are correlated with the patient's HLA II background to some degree, negative staining does not necessarily indicate that the sample came from a non-TB patient; however the sample may be from an TB patient with an unmatched HLA II background. The same level of tetramer staining reflects different amounts of the peptide presenting to the APCs due to the different HLA backgrounds of the patients. This HLA II restriction may be detrimental to the successful and extensive use CD4^+^ TCR tetramers, but perhaps we can solve the problem by mixing different MTB peptide-specific tetramers that match different HLA-DR backgrounds in a single reagent or arrange them into a protein array in order to reduce the false-negative rate. The exquisite binding sensitivity and specificity exhibited by these multimeric TCRs allows us to monitor quantitative modifications in the antigens displayed on the APCs and investigate the binding parameters of TCRs with cross-reactive HLA-DR. We carried out a small-scale study that consisted of monitoring PLF and cerebrospinal fluid (CSF) samples from patients with tuberculous pleuritis and tuberculous meningitis, respectively, using eu- and hu-tetramer stainings and flow cytometry, but were unsuccessful due to very low ratios or low numbers of monocytes and macrophages in these samples (data not shown). Perhaps this problem can be solved by using a Ficoll-Hypaque density gradient, slide smears, and staining. Further studies are needed to obtain additional information about that how many HLA-DR-restricted TCR tetramers can bind with peptide/HLA-DR, the affinities between TCR tetramers and the different forms of HLA-DR, how *in vivo* dynamic changes in APCs are related to TB infection and latent TB infection, and the effects of anti-TB treatment.

Our data are the first description of MTB-specific human CD4^+^ TCR tetramers. These soluble CD4^+^ TCR tetramers demonstrate great diagnostic potential and provide valuable insights into the mechanisms of antigen presentation and its relationship with TB infection and latent TB infection, and can potentially be used to develop revolutionary immunotherapies by enabling the targeted delivery of drugs.

## Materials and Methods

### Ethics statement

The collection, delivery use of clinical samples obtained from TB patients and other control donors and the experimental procedures were approved by the Medical Ethics Committee of Zhongshan School of Medicine, Sun Yat-sen University, the Biosafety Management Committee of Sun Yat-sen University, and the Medical Ethics Committee of Guangzhou Chest Hospital, respectively. All of the patients and healthy donors gave written, informed consent before enrollment in this study.

### Patient recruitment and sample collection

Patients were recruited from the Guangzhou Chest Hospital, Guangzhou, China between October 2009 and February 2011. A diagnosis of active TB was made based on the following: (1) positive sputum smear or culture results for MTB; (2) the detection of active PTB lesions or extrapulmonary TB lesions by X-ray examination or the detection of active PTB or lymph node TB in tissue sections by MTB antigen-specific immunohistochemistry; and (3) the presence of typical symptoms such as cough, expectoration, bloody sputum or hemoptysis, chest distress, chest pain, short breath, and lymphadenovarix. The possibility of malignant lesions in the lung or lymph node sections was ruled out using these criteria. PBL samples, PLF samples, and frozen 8-µm-thick sections of lung and lymph node granuloma and cavernous tissues were obtained from the inpatients, who were either untreated or in the initial stages of treatment (within the first 20 days), and used in this study, in addition to PBL samples that were collected from PTB outpatients during TB development and treatment.

The PBL samples from the non-PTB patients and tissue sections from non-TB patients with pulmonary or lymph node infections were collected from the Guangzhou Chest Hospital. PBL samples from healthy donors and umbilical cord blood samples were collected from the Guangzhou Blood Center and Guangzhou Women and Children's Medical Center, respectively, and used as the study controls.

### Obtaining MTB antigen-specific CD4^+^ T cells

The IFN-γ-ELISPOT assay was used to screen for CD4^+^ T cells that were secreting IFN-γ in response to MTB-specific peptides E6, E7, C5, and C14 in PLF samples obtained from patients with tuberculous pleuritis, as described in previous studies [20], [57]. Briefly, MultiScreen ELISPOT plates (Millipore, Bedford, MA, USA) were coated with 5 µg/mL mouse anti-human IFN-γ capture antibody (eBioscience, San Diego, CA, USA) and stored at 4 °C overnight. After blocking, 2.0–5.0×10^5^ mononuclear cells from the PLF samples were co-incubated with the peptide at a final concentration of 10 µg/mL in a total volume of 200 µL per well for 16–18 hours in the culture medium (complete RPMI with 10% fetal calf serum [FCS; Hyclone, Logan, UT, USA]). After washing, the wells were incubated with 250 µg/mL biotinylated mouse anti-human IFN-γ antibody (eBioscience) for 2 hours at room temperature. After washing again, the plates were incubated with 1∶10000 diluted streptavidin-conjugated alkaline phosphatase (AP) (Pierce, Rockford, IL, USA) for 2.0–2.5 hours. IFN-γ-specific spots were developed by adding BCTP/NBT solution (Pierce) into each well after washing. The reaction was stopped after 15 minutes by rinsing the wells with distilled water. Spots were counted using an ELISPOT reader (Cellular Technology Ltd., Shaker Heights, OH, USA).

The CD4^+^ T cells were separated from the ELISPOT-positive PLF mononuclear cells using immunomagnetic anti-human CD4 particles-DM beads (BD Biosciences, Franklin Lakes, CA, USA), according to the manufacturer's protocol, and resuspended in RPMI-1640 medium at a concentration of 1.0×10^7^ cells/mL. CFSE (Enzo Life Sciences, Lausen, Switzerland) was added to the cell suspension to reach a final concentration of 5 µM. The cell suspension was incubated for 10 minutes at room temperature in the dark. Labeling was terminated by adding the same volume of 100% FCS in order to quench the free CFSE for 10 minutes at room temperature. The labeled cells were washed 3× with sterile phosphate buffer saline (PBS) containing 10% FCS, and then counted. Approximately 2.0–5.0×10^6^/mL cells were seeded into 1.5 mL of culture media (complete RPMI with 10% FCS) that was supplemented with 10 µg/mL of the specific peptide and recombinant interleukin (rIL)-2 (30 IU/mL; PeproTech, Rocky Hill, NJ, USA) per well in 24-well tissue culture plates (Becton Dickinson, San Jose, CA, USA), then incubated at 37 °C in an atmosphere of 5% CO_2_ in order to allow the peptide-specific CD4^+^ T cells to expand. Two days later, the cells were administered 30 IU/mL rIL-2 and 10 µg/mL of the same peptide every 2 days for 9 days, then harvested and stained with PE-labeled mouse anti-human CD4 antibody (CD4-PE; Ancell, Bayport, MN, USA). Finally, the peptide-responsive and expanded CD4^+^ T cells were sorted using a Coulter EPICS ALTRA cell sorter (Beckman Coulter, Fullerton, CA, USA). The purity of the cells was always >98%.

### Establishment of stable cell lines expressing soluble TCR heterodimers

Total RNA was extracted from the peptide-responsive CD4^+^ T cells using the TRIzol one-step method, then reverse transcripted to cDNA using the BD SMART Polymerase Chain Reaction (PCR) cDNA Synthesis Kit (BD Biosciences). The complete code sequences of TCR α and β chains were amplified using the multiplex PCR method and different primer groups. The specific product was purified, digested with restriction enzymes, and ligated using the pGEM-7Zf (+) vector (Promega, Madison, WI, USA), then transferred to competent *Escherichia coli* DH5α cells. Positive recombinant clones were screened and identified by restriction-endonuclease digestion and sequencing. The frequencies of the TCR VA/VB and JB subfamilies and CDR3 sequences were analyzed using software provided with the International ImMunoGeneTics Information System® (IMGT/V-QUEST, http://www.imgt.org/IMGT_vquest/share/textes/). TCR α and β chains containing high-frequency CDR3 sequences were selected to construct the TCR tetramers.

In order to construct the soluble biotinylated TCR molecules in the expressed cell lines, the transmembrane domain and intracellular fragments of TCR α and β chains were cut and the remaining parts were inserted into FB-pGEM-7Zf (+) or JB-pGEM-7Zf (+) vectors, respectively. These vectors were obtained in previous studies and contain complementary, hydrophilic, and polar amino acid fragments (Fos and Jun) and the BirA-dependent biotinylation substrate peptide (BSP). TCRα-FB-pGEM-7z (+) and TCRβ-JB-pGEM-7zf (+) plasmids were constructed.

The modified TCR α and β chains, which have the same CDR3 domain and amino acid sequences in many CD4^+^ T-cell clones, were chosen and subcloned into a eukaryotic expression vector, pMT/V5-His (Invitrogen, Carlsbad, CA, USA), which consisted of a metal promoter, multiple cloning sites, the V5 epitope, and 6-histidine tags. pMT-TCRα, pMT-TCRβ, pMT/Bip-BirA, and pCoHygro (7∶7∶1∶1 molar ratio) were cotransfected into S2 cells (Invitrogen) by calcium phosphate precipitation. The pMT/Bip-BirA plasmid contains the BirA gene that encodes biotin-protein ligase (BirA enzyme). The pCoHygro vector (Invitrogen) expressing hygromycin was used to select stable transfected cell lines.

Cells were cultured at 28 °C overnight in Schneider's Drosophila medium (Invitrogen) containing 270 µg/mL hygromycin-B and 10% FCS. After 4–5 weeks of hygromycin-B selection, a stable co-transfected cell line was established. Then, limited dilution cultivaions were processed until a stable monoclonal cell line was obtained, which was identified by PCR amplification of the sequences of TCR α, TCR β, and BirA carried by the corresponding recombinant vectors. The following specific primers of the C-region of TCR and BirA were used as follows: forward primer, 5′-ACTGTGCTAGACATGAGGTC-3′, and reverse primer, 5′-CGCGTCGACTGACAGGTTTTGA-3′, for the TCR α chain; forward primer, 5′-AGATACTGCCTGAGCAG-3′, and reverse primer, 5′-CGCGTCGACCTCATAGAGGATG-3′, for the TCR β chain; and forward primer, 5′-AAGGATAACACCGTGC-3′, and reverse primer, 5′-TTATTTTTCTGCACTACGCA-3′, for BirA.

### Expression and purification of TCR heterodimers

The selected stable S2 cell line that was cotransfected with pMT-TCRα, pMT-TCRβ, pMT/Bip-BirA, and pCoHygro was added to serum-free Schneider's Drosophila medium containing 160 µg/mL hygromycin-B. The expanded cells were transferred to 1.0-L conical flasks (Wheaton, Millville, NJ, USA) for large-scale culturing at 28 °C, placed on a 120–140-rpm rotary shaker, and secretory expression was induced using 500 mM CuSO4. Heterodimers were directly biotinylated by cell cultivation using 1.0 µg/mL d-biotin for 72 hours. The clarified supernatants were collected by centrifugation. Free Cu^2+^ and other impurities in the medium were removed and the proteins were precipitated from the supernatant by adding polyethylene glycol 6000 (PEG 6000; Sigma-Aldrich, St. Louis, MO, USA) until the final concentration was 12%. The sediment was resuspended by constant stirring in PBS (pH 8.0) containing 10 mM imidazole. The sample was applied to a Ni-NTA agarose affinity column (Qiagen, Valencia, CA, USA), washed with PBS (pH 8.0) containing 20 mM imidazole, and then recombinant biotinylated proteins with 6-histidine tags were eluted out using 250 mM imidazole. After dialyzation with PBS (pH 8.0), the purified samples were concentrated by ultrafiltration using a 30-kDa molecular weight cutoff concentrator (Millipore). At each purification step, small aliquots were subjected to SDS-PAGE, dot blot and Western blot assays. Protein concentrations were determined using the Coomassie brilliant blue method.

### Identification of biotinylated TCR heterodimers

For the dot blot assay, the cultured supernatant or the purified solution was bound to polyvinylidene fluoride (PVDF) membranes (Bio-Rad, Hercules, CA, USA). The membrane was blocked overnight at 4 °C in PBS containing 5% skim milk, then incubated overnight at 4 °C with a 1∶3000 dilution of the anti-TCR α/β antibody (eBioscience) or anti-V5 antibody (BD Pharmingen), After washing with PBS containing 0.05% Tween 20 (PBST), the membrane was incubated with AP-conjugated goat anti-mouse IgG antibody (1∶3000 dilution; Bethyl, Montgomery, TX, USA) for 1 hour at room temperature. After intensive washing with PBST, the membrane was developed using BCIP/NBT solution for 15 minutes. To confirm biotinylation of the expressed TCR monomers, another blocked membrane was incubated with streptavidin-AP (1∶10000 dilution) for 1 hours and developed using the BCIP/NBT solution.

For SDS-PAGE, the purified protein samples in Ni-NTA agarose were run in 12% SDS-PAGE under denaturing conditions and stained with coomassie blue (Bio-Rad) in order to calculate the molecular weight of the protein by comparing its migration rate with that of standard protein markers. The expression and purified efficiency of recombinant TCR α, TCR β, and their heterodimers were determined by observing the bands.

For the Western blot assay, proteins from the cultured supernatants or the purified solution were separated on 5–12% SDS-PAGE gels and transferred to PVDF membranes using Trans-Blot SD Cell (Bio-Rad). Membranes were blocked overnight at 4 °C in PBS containing 5% skim milk. The TCR α/β chain was identified by overnight incubation in a 1∶3000 dilution of the anti-TCR α/β antibody in PBS at 4 °C. The membranes were washed 3× (5 minutes per wash) in PBST and incubated for 1 hour with AP-conjugated goat anti-mouse IgG antibody in PBS at 1∶3000 dilution. Then, the membranes were washed 5× and detection was performed using the BCIP/NBT solution for 15 minutes.

### Preparation of TCR tetramers

The purified biotinylated TCR monomers were mixed with one-eighth of the molar amount of streptavidin by repeatedly adding streptavidin 8×, and each reaction mixture was incubated for 5 minutes at room temperature. Subsequently, the final reaction mixture was incubated for 30 minutes at room temperature, purified, and concentrated by ultrafiltration and buffer exchange with PBS using a 100-kDa molecular weight cut-off concentrator (Millipore). For staining and flow cytometric analysis of the PBL samples, the purified biotinylated TCR monomers were tetramerized with PE-conjugated streptavidin (eBioscience); as well as with pure streptavidin (eBioscience) for proliferation and blocking expansion assays of CD4^+^ T cells and with FITC-conjugated streptavidin (eBioscience) for *in situ* staining of the tissue sections, respectively.

### Determination of the affinities of the TCR tetramers to different HLA-DR allele molecules

To compare the binding affinities of the TCR tetramers to specific HLA class II molecules, the S2 cell lines expressing different MTB peptide/HLA-DR molecules on the cell membranes (using the previously constructed artificial APCs) were cultured and screened for CD4^+^ TCR tetramers by flow cytometry. After 48 hours of induction by CuSO_4_, the cells were incubated with streptavidin to block endogenous biotin, and then stained with the PE-labeled TCR tetramer and the FITC-conjugated mouse anti-human HLA-DR antibody (L243-FITC; BD Pharmingen) at 4 °C for 20 minutes before beginning flow cytometric analysis. S2 cells expressing only HLA-DR and non-induced S2 cells served as the controls.

### Peptide-stimulated proliferation and TCR tetramer-blocking expansion assays of CD4^+^ T cells collected from clinical blood samples

PBMC were isolated from the PBL samples obtained from the PTB patients and resuspended in RPMI-1640 medium at a concentration of 1.0×10^7^ cells/mL. CFSE-labeled cells were stained and counted as described above. Approximately 3.0–5.0×10^5^ cells/mL were seeded in 0.2 mL of culture media (complete RPMI with 10% FCS) per well in 96-well tissue culture plates (Becton Dickinson) in the presence of 10 µg/mL of the specific peptide in order to expand the peptide-specific lymphocytes. Peptide-specific expansion was performed either in the presence or absence of a single dose of the non-labeled TCR tetramer (1 µg) and cultured for 9 days in complete RPMI medium containing 30 IU/mL IL-2. Parallel lymphocytic expansion in the presence of an unrelated peptide (oncopeptide) was also concurrently monitored to determine the blocking specificity of the TCR tetramer. Peptide-specific and TCR tetramer-blocking expansion of the CD4^+^ T cells were monitored by CD4-PE (Ancell) staining and flow cytometric analysis.

### Detection of tetramer-bound CD14^+^ monocytes in clinical blood samples by tetramer staining and flow cytometric analysis

The tetramers were routinely titrated in order to obtain the optimal concentration for nonspecific binding. Antibodies and tetramers were added to freshly isolated PBMCs from PTB patients at different stages and control donors under optimized conditions: 1.0×10^6^ mixed cells were stained with 1.25 µg of PE-labeled TCR tetramer in 80 µL PBS for 20 minutes at 4 °C in the presence of 1.3 µL FITC-labeled mouse anti-human CD14 antibody (CD14-FITC; Ancell). The stained samples were analyzed using a Coulter EPICS XL-MCL flow cytometer (Beckman Coultronics, Margency, France) and FCS Express V3 software (De Novo, Los Angeles, CA, USA).

### The analysis of MTB-induced apoptosis of monocytes and the *in vitro* protective effects of INH

THP-1 cells were co-cultured with 3×10^5^ MTB H37Ra. A single dose of INH (Sigma-Aldrich; 30 µg/mL, 150 µg/mL and 250 µg/mL, respectively) was added. Apoptotic cells were observed by annexin-V-FITC/PI (BD Pharmingen) staining and flow cytometric analysis at 24 and 48 hours.

### Analysis of early-apoptotic of CD14^+^ cells in blood samples from healthy donors, and untreated and treated TB patients

CD14^+^ cells obtained from PBL samples from healthy donors, and untreated- and treated- (<5 days, 15–30 days and >30 days from the initiation of treatment) TB patients, were sorted using CD14 immune magnetic beads (Miltenyi Biotec, Freiburg, Germany), and early apoptosis was immediately detected using annexin-V-FITC/PI staining and flow cytometric analysis as described above.

### *In situ* detection of tetramer-bound CD14^+^ macrophages in tissue sections by tetramer immunohistochemical staining

Two staining strategies were used to verify the specific binding affinities of the TCR tetramers. The first staining strategy was to identify the location of the MTB specific antigen and tetramer-positive cells in the lung and lymph node sections from TB patients; tissue sections from non-TB patients with pulmonary or lymph node infections were used as controls. Briefly, frozen sections were fixed in 4% polyformaldehyde, washed with PBST, and blocked using PBS containing 2% BSA or the avidin/streptavidin-biotin blocking kit for 30 minutes at room temperature, respectively. Then the sections were incubated in a moist container at 4 °C overnight with 50 µL of the FITC-labeled TCR tetramer (final concentration: 10 µg/mL) and rabbit anti-MTB antibody (1∶300 dilution; Abcam, Cambridge, United Kingdom), followed by incubation with mouse anti-FITC antibody (1∶300 dilution; Sigma-Aldrich) for 30 minutes at room temperature. After washing with PBST to remove the unbound primary antibodies, the sections were incubated with Alexa 555-labeled goat anti-rabbit antibody (1∶2000 dilution; Invitrogen) and Alexa 488-labeled goat anti-mouse antibody (1∶2000 dilution; Invitrogen) for 1 hour at room temperature, then incubated with 1∶3000 DAPI for 5 minutes at room temperature. After washing with PBST, the sections were observed *in situ* using confocal laser-scanning microscopy to determine the distribution of the MTB-specific antigen cells that were bound to the tetramer.

The second staining strategy was to determine if the tetramer-positive cells were APCs. The anti-CD14 antibody was used to identify monocytes and macrophages in fresh-frozen 8-µm-thick sections of lung and lymph node tissues from TB patients; the same controls described above were also used. Briefly, frozen sections were fixed in 4% polyformaldehyde, washed with PBST, and blocked with PBS containing 2% BSA or the avidin/streptavidin-biotin blocking kit for 30 minutes at room temperature, respectively. Then, the sections were incubated in a moist container at 4 °C overnight with 50 µL FITC-labeled TCR tetramer (10 µg/mL) followed by the rabbit anti-FITC antibody (1∶300 dilution: Invitrogen) for 30 minutes. CD14 was detected following incubation with mouse anti-human CD14 MAb (1∶200 dilution; Invitrogen) for 1 hour. After removing the unbound primary antibodies and washing with PBST, the sections were incubated with Alexa 555-labeled goat anti-rabbit antibody (1∶2000 dilution) and Alexa 488-labeled goat anti-mouse antibody (1∶2000 dilution) for 1 h, then incubated with DAPI (1∶300 dilution) for 5 minutes at room temperature. After washing with PBST, the sections were monitored *in situ* using confocal laser-scanning microscopy to determine the distribution of the tetramer-bound CD14^+^ macrophages.

### HLA genotyping of the HLA-DR alleles obtained from TB patients

Clinical samples from the TB patients with high-affinity reactions to the TCR tetramers and samples from donors with negative reactions to the TCR tetramers on double-label staining were chosen for HLA genotyping. DNA was extracted from whole blood samples using the QIAamp DNA kit (Invitrogen). The HLA type of each individual was determined by sequence-specific primer PCR (SSP-PCR) according to manufacturer's protocol.

### Statistical analysis

Data were statistically analyzed by using SPSS software (version 16.0 for Windows; SPSS, Inc., Chicago, IL, USA). Abnormally distributed data obtained from the flow cytometric analyses were presented using median values and the 25^th^ and 75^th^ percentiles. The results were compared using the Kruskal-Wallis H test for multiple (i.e., ≥3) independent samples, while the Mann-Whitney U test was used to compare 2 independent samples. Any *p*-values<0.017 and <0.05 were considered statistically significant, respectively.

## Supporting Information

Figure S1 **Representative FACS dot plots of the binding of TCR tetramers to artificial APCs.** Artificial APCs were stained with TCR tetramer and anti-HLA-DR antibody (L243-FITC). Only background stainings of the tetramers were seen in non-induced cells (0.32% in C14/HLA-DRB1*150101 stained with eu and 0.23% of C14/HLA-DRB1*08032 stained with hu) or cells expressing only HLA-DR without the peptide (0.18% of HLA-DRB1*150101 stained with eu and 0.16% of HLA-DRB1*08032 stained with hu). After induction, positively stained cells expressing C14/HLA-DRB1*150101 with eu (10.90%) or C14/HLA-DRB1*08032 with hu (3.13%), as well as negatively stained cells expressing E7/HLA-DRB1*1504 with eu (0.46%) or C5/HLA-DRB1*1503 with hu (0.67%), were observed.
(TIF)
