# Supplementary figures and images for: Relatively Low Level of Antigen-specific Monocytes Detected in Blood from Untreated Tuberculosis Patients Using CD4+ T-cell Receptor Tetramers
Source: PLoS Pathog. 2012 Nov 29;8(11):e1003036. doi: 10.1371/journal.ppat.1003036 (PMC3510242; doi:10.1371/journal.ppat.1003036)

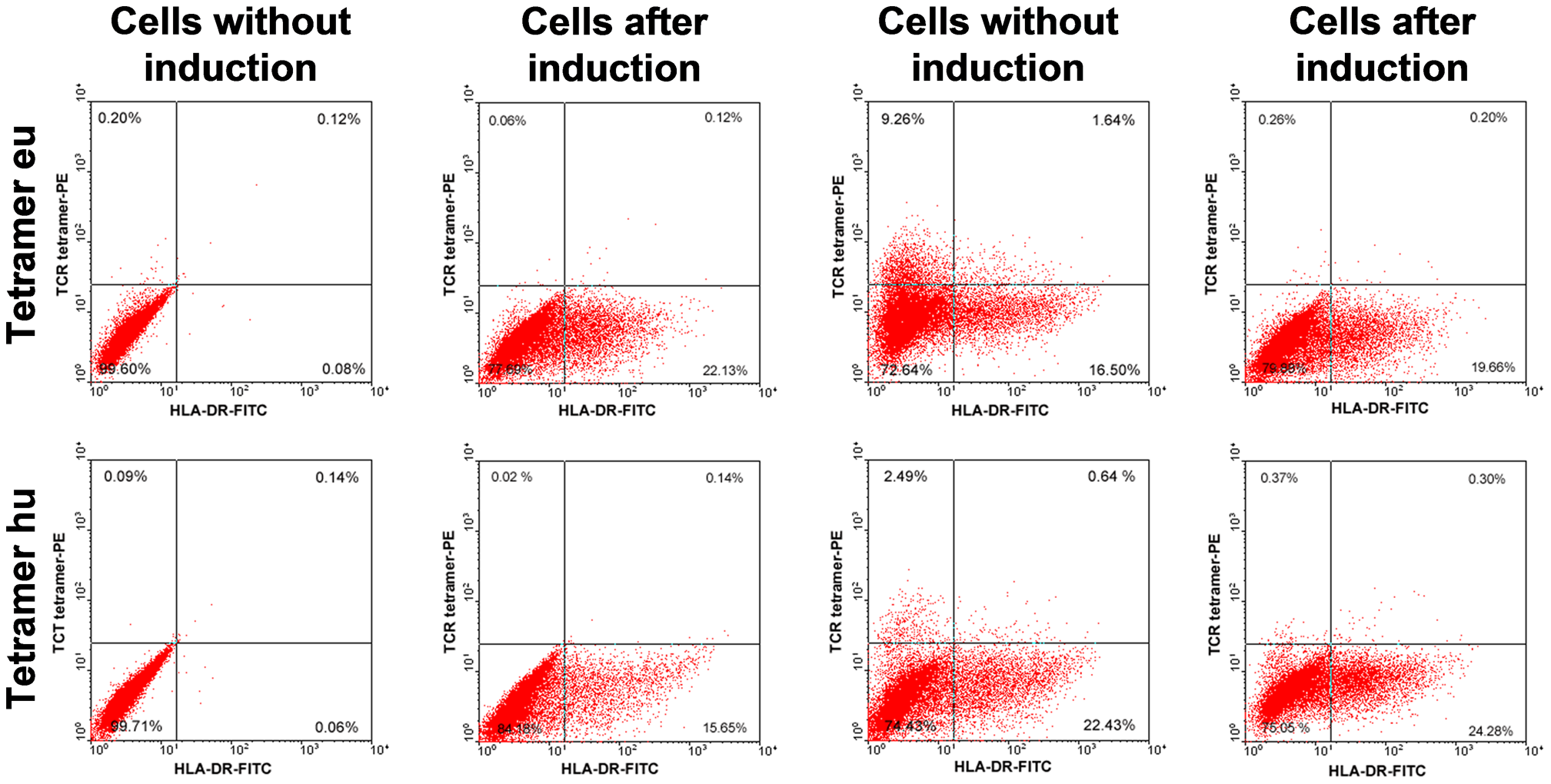

Supplement: Figure S1 — Representative FACS dot plots of the binding of TCR tetramers to artificial APCs. Artificial APCs were stained with TCR tetramer and anti-HLA-DR antibody (L243-FITC). Only background stainings of the tetramers were seen in non-induced cells (0.32% in C14/HLA-DRB1*150101 stained with eu and 0.23% of C14/HLA-DRB1*08032 stained with hu) or cells expressing only HLA-DR without the peptide (0.18% of HLA-DRB1*150101 stained with eu and 0.16% of HLA-DRB1*08032 stained with hu). After induction, positively stained cells expressing C14/HLA-DRB1*150101 with eu (10.90%) or C14/HLA-DRB1*08032 with hu (3.13%), as well as negatively stained cells expressing E7/HLA-DRB1*1504 with eu (0.46%) or C5/HLA-DRB1*1503 with hu (0.67%), were observed. (TIF) [file ppat.1003036.s001.tif]
